# Transparent Platinum Counter Electrode Prepared by Polyol Reduction for Bifacial, Dye-Sensitized Solar Cells

**DOI:** 10.3390/nano10030502

**Published:** 2020-03-11

**Authors:** Alvien Ghifari, Dang Xuan Long, Seonhyoung Kim, Brian Ma, Jongin Hong

**Affiliations:** Department of Chemistry, Chung-Ang University, 84 Heukseok-ro, Dongjak-gu, Seoul 06974, Korea; alvien.ghifari@gmail.com (A.G.); dxlong.bk@gmail.com (D.X.L.); kimsh9560@gmail.com (S.K.); kehgeeng@gmail.com (B.M.)

**Keywords:** dye-sensitized solar cell, counter electrode, bifacial, platinum, ethylene glycol

## Abstract

Pt catalytic nanoparticles on F-doped SnO_2_/glass substrates were prepared by polyol reduction below 200 °C. The polyol reduction resulted in better transparency of the counter electrode and high power-conversion efficiency (PCE) of the resultant dye-sensitized solar cells (DSSCs) compared to conventional thermal reduction. The PCEs of the DSSCs with 5 μm-thick TiO_2_ photoanodes were 6.55% and 5.01% under front and back illumination conditions, respectively. The back/front efficiency ratio is very promising for efficient bifacial DSSCs.

## 1. Introduction

Dye-sensitized solar cells (DSSCs) have shown promise as low-cost photovoltaics compared to commercially available Si solar cells. They hold great potential for building-attached photovoltaics (BAPVs) and building-integrated photovoltaics (BIPVs), because of their adjustable color/transparency and superior performance in dim light [1,2,3,4]. Recently, bifacial DSSCs, which can convert incoming sunlight to electricity through both front and back sides, have been an attractive alternative for photovoltaic devices [5,6]. The standard DSSC consists of a substrate coated with transparent conducting oxides (TCOs), a dye-grafted mesoscopic TiO_2_ photoanode, a platinized counter electrode (CE), and an electrolyte containing a redox couple. Among the key components, the CE plays a prominent role in maintaining a flow of current by regenerating the redox mediator. Unfortunately, Pt is a highly expensive and scarce metal, and thus alternative materials, including carbon-based materials [7,8], transition metal compounds [9], conducting polymers [10,11], and their composites [12,13] have been explored.

Nevertheless, Pt is still favored because of its superior electrocatalytic activity and good conductivity. The conventional methods for preparing Pt CEs are vacuum sputtering of a Pt target and thermal decomposition of a platinic acid (H_2_PtCl_6_) precursor [14,15]. However, these high-energy-consuming approaches increase the cost and energy payback time of DSSCs. In addition to logistical issues, the light absorption at the Pt CEs should be minimized for bifacial operation. Therefore, it is crucial to develop new preparation methods for highly transparent Pt CEs at low temperatures.

Polyol-based synthesis is a versatile and straightforward liquid-phase method that uses high-boiling and multivalent alcohols to synthesize nanomaterials without the requirements of high pressure and autoclaves [16,17]. Polyol can simultaneously act as a reducing agent and water-equivalent solvent. Its chelating ability or controlling the nucleation of nanomaterials. For example, Ouyang and coworkers reported nanostructured Pt CEs prepared by polyol reduction of H_2_PtCl_6_ in ethylene glycol (EG) [18,19]. They also utilized EG vapor for solventless chemical reduction of the platinum precursor below 200 °C [20]. Song et al. employed the hydrolysis of urea in a two-step EG solution reduction to achieve uniform dispersion and a smaller size of Pt nanoparticles on conducting glass substrates [21]. Unfortunately, the Pt CEs were prepared by drop-casting the EG-based Pt precursor solution. It has proven challenging to prepare a thin layer of Pt nanoparticles and thus provide highly transparent CEs for the bifacial DSSCs. Therefore, we prepared a highly transparent Pt counter electrode by spin-coating a platinic EG solution and further chemical reduction below 200 °C. We also investigate the photovoltaic performance of the DSSCs from the viewpoint of bifacial operation.

## 2. Materials and Methods

### 2.1. Fabrication of Pt Counter Electrodes

**Figure 1:**
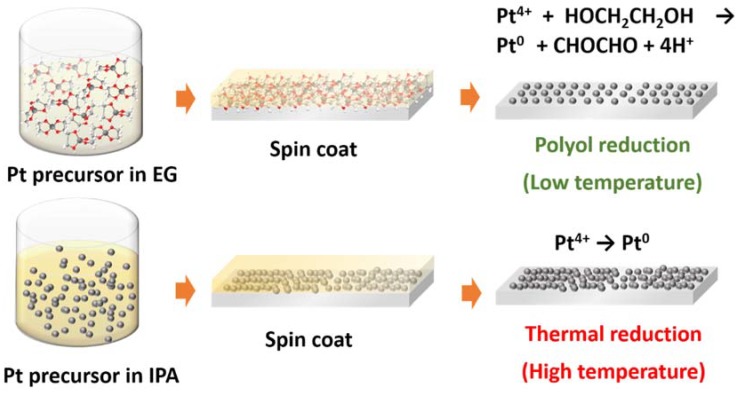
Schematic diagram of counter electrode preparation: polyol reduction (PR) and thermal decomposition (TD).

F-doped SnO_2_ (FTO) glass (TEC 8, Pilkington; sheet resistance = 8Ω/□) substrates were ultrasonically cleaned using acetone, isopropyl alcohol, and deionized water. The substrates were baked at 150 °C for 10 min to completely remove residual water. Then 10 mM platinic acid in ethylene glycol was spin-coated on the FTO substrates. Subsequently, the sample was placed in a muffle furnace and heated to a certain temperature (e.g., 130, 150, 170, 190, and 210 °C). The annealing was maintained for 12 h, and then the sample was cooled down to room temperature. For comparison, 40 mM of the platinic acid solution in 2-propanol was prepared and then spin-coated on the same substrates, followed by heating at 425 °C for 1 h in the muffle furnace [22,23,24]. The schematic diagram of Pt CE preparation is shown in Figure 1.

### 2.2. Characterization

The surface images of the fabricated Pt CEs were obtained using a field-emission scanning electron microscope (FE-SEM; Sigma, Carl Zeiss AG, Oberkochen, Germany). X-ray photoelectron spectroscopy (XPS) spectra were acquired using a Thermo Scientific K-alpha XPS system (Waltham, MA, United States) with an Al Kα X-ray source monochromator (1486.6 eV). The optical transmittance spectra were recorded using UV/Vis spectroscopy (V-730, JASCO, Tokyo, Japan). An electrochemical measurement station (CompactStat, Ivium Technologies, Eindhoven, The Netherlands) was used to conduct all electrochemical characterization. Cyclic voltammetry (CV) was recorded at a scan rate of 50 mV/s with a three-electrode system that consists of a Pt CE as a working electrode, a Pt wire as a counter electrode, and Ag/AgCl as a reference electrode. A solution of 10.0 mM LiI, 1.0 mM I_2_, and 0.1 M LiClO_4_ in CH_3_CN was used as the electrolyte to investigate the electrocatalytic properties of the Pt CEs for redox reactions. Tafel and electrochemical impedance spectroscopy (EIS) measurements were performed on symmetric cells consisting of CE|electrolyte|CE. Tafel polarization measurements were conducted at a scan rate of 10 mV/s. In EIS measurements, a 10 mV amplitude sinusoidal potential perturbation was input over a frequency range from 1 MHz to 0.1 Hz at zero bias potential. The EIS spectra were analyzed using the equivalent circuit fitting routine in the ZView software (AMETEK, Leicester, UK).

### 2.3. Device Fabrication and Characterization

The FTO glass substrates were cleaned with O_2_ plasma for 10 min, dipped in an aqueous solution of 40 mM TiCl_4_ at 75 °C for 30 min, and then rinsed several times with deionized water. The TiO_2_ paste (Transparent TiO_2_, ENBKOREA, Gumi, Korea) was screen-printed on the FTO glass, and the printed film was calcinated at 300 °C for 30 min and 575 °C for 1 h in the muffle furnace. The final areas of the TiO_2_ photoanodes were 0.2025 cm^2^. The photoanodes were treated in the TiCl_4_ solution and then heated at 500 °C for 30 min on a hot plate. After O_2_ plasma treatment, the photoanodes were dipped into a 0.5 mM dye solution of cis-diisothiocyananoto-bis(2,2-bipyridyl-4,4′-dicarboxylate) ruthenium(II) (N719, Merck KGaA, Darmstadt, Germany) in ethanol for 24 h. The dye-grafted photoanode and Pt CE were assembled with 25 µm-thick thermoplastic film (Surlyn, Solaronix, Aubonne, Switzerland) and sealed by heating. An iodide-based redox electrolyte (Iodolyte AN-50, Aubonne, Switzerland) was injected into the pre-drilled holes in the side of the counter electrode and then sealed. The photovoltaic characteristics were investigated under AM 1.5 global one sun illumination (100 mW/cm^2^) using a solar cell I–V measurement system (K3000 LAB, McScience, Suwon, Korea). The photocurrent density (*J_sc_*), open-circuit voltage (*V_oc_*), fill factor (*FF*), and power conversion efficiency (*η*) were recorded simultaneously. Monochromatic incident photon-to-current conversion efficiency (IPCE) was collected to evaluate the spectral response of the solar cells (K3100, McScience, Suwon, Korea). EIS measurement on the fabricated devices was carried out by the same protocol mentioned above.

## 3. Results

**Figure 2:**
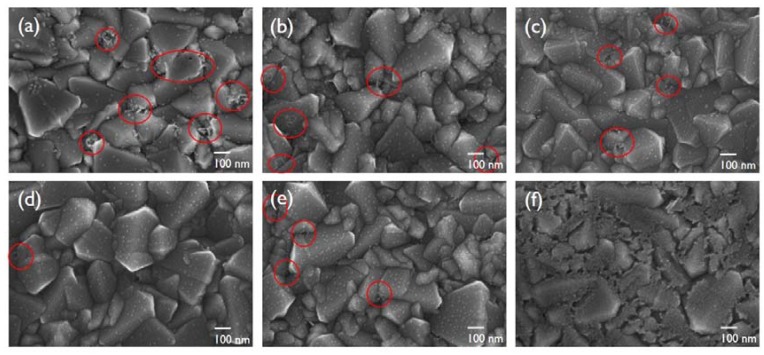
Field-emission scanning electron microscope (FE-SEM) images of Pt films on FTO substrates: (**a**) PR-130, (**b**) PR-150, (**c**) PR-170, (**d**) PR-190, (**e**) PR-210, and (**f**) TD-425.

Polyol-based synthesis is a versatile technique to prepare Pt nanostructures. In this study, EG was chosen as the solvent and reducing agent of Pt precursors because of its low boiling point and viscosity. Also, byproducts from the EG reduction, such as glycoaldehyde and diacetyl, could be easily removed [25]. Figure 2a–f shows the surface of the FTO glass substrates decorated with Pt nanoparticles formed by polyol reduction at different temperatures (130, 150, 170, 190, and 210 °C) and thermal decomposition at 425 °C (hereafter, “polyol-reduced” is abbreviated as “PR” and “thermally decomposed” is abbreviated as “TD”). Tiny Pt nanoparticles (<10 nm) were formed and dispersed on the FTO surface for all reaction temperatures. The chemical reduction by EG allows for depositing Pt nanoparticles at low temperatures, and is thus feasible for plastic substrates. As the reduction temperature increased, the aggregation of the Pt nanoparticles diminished, and no aggregation could be observed at the temperature of 190 °C. This indicates that the slower evaporation of EG could result in the growth of larger nanoparticles, more agglomeration, and dendrites. However, the aggregated nanoparticles appeared again at a temperature higher than the boiling point of EG (i.e., 197 °C) because of the inability to control particle nucleation and growth at the elevated temperature [17]. Also, the pyrolysis of H_2_PtCl_6_ at 425 °C directly led to the formation of large Pt nanoparticles prominently populating the FTO. Accordingly, we predict that their size and distribution will affect the transparency and catalytic activity of the resultant electrodes.

**Figure 3:**
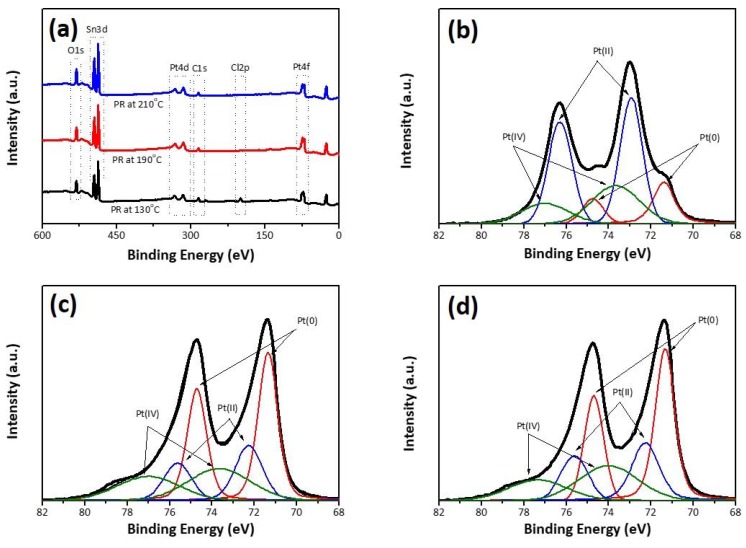
X-ray photoelectron spectroscopy (XPS) scans of polyol-reduced Pt on FTO substrates: (**a**) survey, (**b**) PR-130, (**c**) PR-190, and (**d**) PR-210.

XPS was performed to determine the formation of metallic Pt during our chemical reduction. In Figure 3, the spectra of survey XPS indicates that the samples contained Sn, O, Pt, and Cl—no other elements except carbon were detected. Carbon can result from precursors or sample handling. Interestingly, as the reduction temperature increased, the signal for Cl 2p related to ionic Pt species decreased and then disappeared. Narrow XPS scans of the Pt-4f core level region are also provided in Figure 3. The C 1 s peak at 285.0 eV was used to calibrate all the XPS data. The Pt 4f signal is composed of three pairs of deconvoluted doublets. The first doublet (71.4 eV and 74.8 eV) corresponds to the platinum in the zero-valent state (i.e., Pt(0)), while the second doublet (72.3 eV and 75.6 eV) can be assigned to platinum in the 2+ valence state (i.e., Pt(II)) [26]. The third (73.7 eV and 77.0 eV) is caused by the Pt^4+^ species, such as [PtCl_6_]^2−^, on the surface (i.e., Pt(IV)) [27]. Table 1 summarizes the binding energies and relative integrated peak areas of the deconvoluted peaks at the Pt-4f core level region. The increase in the reduction temperature resulted in the change in the valence state from Pt^2+^ to Pt^0^. After the polyol reaction, the Pt species clustered together with different compositions. Accordingly, the reaction temperature is of significant importance in preparing metallic Pt nanoparticles.

**Figure 4:**
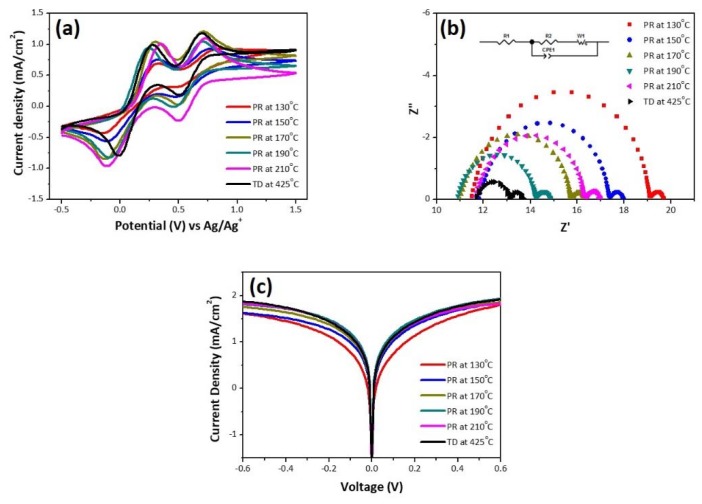
(**a**) Cyclic voltammograms for the redox of $I_{3}^{-}/I^{-}$ species, (**b**) electrochemical impedance spectroscopy (EIS) Nyquist plots for the symmetric cells, and (**c**) Tafel polarization curves.

In the DSSC operation, an $I_{3}^{-}/I^{-}$ redox couple is commonly utilized as a redox mediator. The CE should collect electrons from the external circuit and effectively catalyze the reduction of $I_{3}^{-}$ to $I^{-}$. The electrocatalytic activity of the PR CEs was determined using cyclic voltammetry (CV), as shown in Figure 4a. All CV curves show two typical pairs of oxidation and reduction peaks (Ox-1/Re-1 and Ox-2/Re-2), which are described by Equations (1) and (2), respectively [28]:

(1) $$I_{3}^{-}+2e^{-}\;⇌\;3I^{-}$$

(2) $$3I_{2}+2e^{-}\;⇌\;2I_{3}^{-}$$

The catalytic reduction activity of the CE can be determined by the first oxidation and reduction peaks (Ox-1 and Re-1). Its electrochemical reversibility can be assessed from the peak-to-peak separation (Δ*E_p_*), which is the difference between the anodic and cathodic peak potentials. A smaller Δ*E_p_* reflects the higher electrocatalytic activity of the CE.

EIS measurements were also conducted using the symmetrical cells constructed with two identical electrodes. The Nyquist plots (Figure 4b) consist of two apparent semicircles at a high frequency (first semicircle) and a low frequency (second semicircle), respectively. The Randles-type circuit (insert in Figure 4b) was used to simulate the plots. The corresponding parameters are summarized in Table 2. *R_s_* is the series resistance, and can be derived by the intercept of the high-frequency semicircle on the real axis (*Z*’ axis). *R_ct_* is the charge transfer resistance at the interface between CE and electrolyte, and can be determined from the radius of the first semicircle on the real axis. All the PR-CEs have nearly the same *R_s_*, and thus its effect on photovoltaic performance can be ignored. A smaller *R_ct_* accelerates triiodide reduction, and thus the PR-190 CE has the superior catalytic activity. The low-frequency semicircle results from Nernst diffusion impedance (*Z_N_*) of the redox species in the electrolyte, which is inversely proportional to the diffusion coefficient of $I_{3}^{-}$ in the cells [28]. The decrease in *Z_N_* leads to an increase in the electrocatalytic activity of the CE.

Figure 4c shows Tafel polarization plots of the symmetrical cells comprising the Pt CEs and $I_{3}^{-}/I^{-}$ electrolytes. The Tafel plot can be separated into three consecutive zones: polarization, Tafel, and limit diffusion zones. The Tafel and limit diffusion zones are valuable for obtaining both the limiting current density (*J_lim_*) and current density (*J*_0_), which correlate with the electrocatalytic activity of the CEs [29]. The intersection of the cathodic branch and the equilibrium potential line can be considered *J*_0_, and thus the steep Tafel slope implies a large *J*_0_. Theoretically, *J*_0_ can also be calculated using Equation (3):

(3) $$J_{0}=\frac{RT}{nFR_{ct}}$$

where *R* is the gas constant, *T* is the absolute temperature, *n* is the number of electrons participating in the electrochemical reduction of $I_{3}^{-}$, and *F* is Faraday’s constant. In the limit diffusion zone, *J_lim_* can be determined by the intersection of the cathodic branch and the *y*-axis. *J_lim_* is directly proportional to a diffusion coefficient of $I_{3}^{-}$ (*D*) at the same potential, and can be expressed as Equation (4):

(4) $$J_{lim}=\frac{2neDCN_{A}}{l}$$

where *e* is the elementary charge, *C* is the concentration of $I_{3}^{-}$, $N_{A}$ is the Avogadro constant, and *l* is the distance between two electrodes. Notably, the values of *J*_0_ and *J_lim_* followed the same trend observed in both CV and EIS analyses. Accordingly, the electrocatalytic activity of $I_{3}^{-}/I^{-}$ is as follows: PR-190 > PR-170 > PR-210 > PR-150 > PR-130.

**Figure 5:**
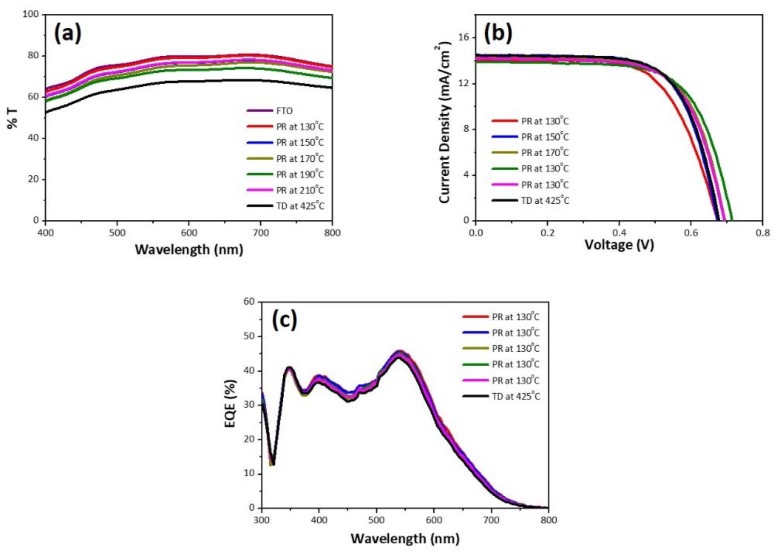
(**a**) Transmittance spectra of the Pt counter electrodes, (**b**) J–V characteristic curves of the dye-sensitized solar cells (DSSCs) (5 μm-thick TiO_2_) under one sun front illumination, and (**c**) their incident photon-to-current conversion efficiency (IPCE) spectra.

The light absorption at the counter electrode should be minimized to improve light harvesting in the bifacial DSSCs. Figure 5a shows the transmittance spectra of the prepared Pt CEs and bare FTO glass. Polyol reduction manifested higher transparency compared to thermal decomposition in the whole visible light regime, which will be beneficial to bifacial applications. Unfortunately, the increase in reduction temperature resulted in the decrease in transmittance of PR CEs. We think that metallic Pt nanoparticles possibly have a negative effect on the light transparency. Figure 5b,c shows, respectively, the current density-voltage (J–V) characteristics and IPCE spectra of the DSSCs (5 μm-thick TiO_2_), which were illuminated from the TiO_2_ photoanode side (i.e., front illumination). The photovoltaic parameters of the front- and rear-illuminated DSSCs are summarized in Table 3. The improved electrocatalytic activity of the PR–Pt CE resulted in better photovoltaic performance: power conversion efficiency (*η*) increased from 5.50% (PR-130) to 6.55% (PR-190). Also, PR-190 exhibited better photovoltaic performance than TD-425 because of the improvement of *R_s_* and *Z_N_* related to the electrocatalytic activity of the CE. It should be noted that all the DSSCs employing PR–Pt CEs maintained more than 76% of their front-illumination efficiency when lit from the Pt CE side (i.e., back illumination). The decrease of *η* in the back-illumination condition is related to the transmission losses due to the Pt-based electrocatalyst and the $I_{3}^{-}/I^{-}$ electrolyte [6]. The ratio of the back-illumination efficiency to the front-illumination efficiency (*η*(*R*)) followed the trend of the transmittance of the PR–Pt CEs observed above.

To date, various transparent CEs have been developed for bifacial applications, such as photovoltaic windows and façades. Table 4 shows the photovoltaic performance of the bifacial DSSCs with platinum and non-platinum CE materials compared to our work. Although DSSC fabrication conditions (e.g., TiO_2_ thickness, dyes, electrolytes) are not the same, our polyol reduction technique proved sufficient for fabricating bifacial DSSCs.

## 4. Conclusions

In this work, highly transparent Pt CEs were prepared using the polyol reduction technique at low temperatures (<200 °C). Our facile and versatile technique provided better electrocatalytic activity and transparency than conventional thermal decomposition methods, and thus brought significant improvement to the photovoltaic performance of the bifacial DSSCs. In particular, the bifacial DSSC with PR-190 attained 6.55% for front illumination and 5.01% for back illumination, while that with TD had 6.42% for front illumination and 4.35% for back illumination. Because our process does not require an elevated temperature, we are currently exploring the fabrication of flexible bifacial DSSCs that employ polymeric substrates. In addition, further optimization (e.g., [Co(bpy)_3_]^3+/2+^ electrolytes) should result in better bifacial DSSC performance.

## Figures and Tables

**Table 1:** Binding energies (eV) and relative proportions (S, in %) of each component measured by XPS.

	Pt(0)			Pt(II)			Pt(IV)		
	4f_5/2_	4f_7/2_	S	4f_5/2_	4f_7/2_	S	4f_5/2_	4f_7/2_	S
PR-130	74.8	71.4	23.7	76.3	72.9	38.8	77.0	73.7	37.5
PR-190	74.7	71.4	48.4	75.6	72.3	22.7	77.0	73.7	28.9
PR-210	74.7	71.4	44.8	75.6	72.3	20.9	77.0	73.7	34.3

**Table 2:** Electrochemical parameters of polyol-reduction Pt films at different reaction temperatures.

No	Δ*E_p_* (V)	*J*_0_ (mA/cm^2^)	*J_lim_* (mA/cm^2^)	*R_s_* (Ω/cm^2^)	*R_ct_* (Ω/cm^2^)	*Z_N_* (Ω/cm^2^)
PR-130	0.47	4.99	0.21	8.05	5.19	0.44
PR-150	0.44	9.16	0.21	8.26	3.79	0.44
PR-170	0.43	11.14	0.24	7.69	3.21	0.41
PR-190	0.35	14.76	0.26	7.63	2.23	0.44
PR-210	0.46	12.44	0.26	8.13	3.17	0.48
TD-425	0.29	11.62	0.27	11.79	1.27	0.59

**Table 3:** Photovoltaic parameters of DSSCs (5 μm-thick TiO_2_) with different Pt counter electrodes ^a^.

No	Illumination	*V_oc_* (V)	*J_sc_* (mA/cm^2^)	*FF* (%)	*η* (%)	*η* (*R*) (%)
PR-130	Front	0.666 ± 0.007	13.82 ± 0.23	59.70 ± 5.48	5.50 ± 0.66	81.03 ± 1.68
	Back	0.664 ± 0.008	10.67 ± 0.32	62.74 ± 4.85	4.45 ± 0.50	
PR-150	Front	0.673 ± 0.003	13.99 ± 0.51	67.45 ± 1.43	6.35 ± 0.33	77.57 ± 1.57
	Back	0.670 ± 0.004	10.65 ± 0.62	69.04 ± 1.22	4.93 ± 0.35	
PR-170	Front	0.694 ± 0.006	13.96 ± 0.31	67.20 ± 1.33	6.51 ± 0.30	76.73 ± 1.33
	Back	0.689 ± 0.003	10.62 ± 0.52	68.28 ± 0.90	5.00 ± 0.30	
PR-190	Front	0.702 ± 0.011	13.77 ± 0.12	67.66 ± 1.55	6.55 ± 0.28	76.61 ± 3.24
	Back	0.699 ± 0.00	10.44 ± 0.23	68.71 ± 1.76	5.01 ± 0.24	
PR-210	Front	0.695 ± 0.004	13.76 ± 0.45	67.02 ± 0.84	6.41 ± 0.29	76.70 ± 2.14
	Back	0.692 ± 0.008	10.42 ± 0.68	68.22 ± 0.94	4.92 ± 0.3	
TD-425	Front	0.682 ± 0.00	13.97 ± 0.46	67.36 ± 0.72	6.42 ± 0.28	67.92 ± 2.66
	Back	0.672 ± 0.005	9.40 ± 0.16	68.97 ± 0.66	4.35 ± 0.05	

^a^ The parameters were obtained from at least five cells fabricated with each CE condition; each cell was measured five times.

**Table 4:** Photovoltaic parameters of bifacial DSSCs with different CE materials.

Material	Method	T (%)	*V_oc_* (V)	*J_sc_* (mAcm^−2^)	*FF* (%)	*η (front)* (%)	*η (back)* (%)	*η (R)* (%)	Refs.
PEDOT	Electropolymerization	~90 ^a^	0.751	14.60	67	7.40	5.23	70.7	[6]
Carbon	Carbonization	~75	0.721	10.52	60	6.07	5.04	83.0	[7]
Ni_3_S_4_	Hydrothermal	~70	0.700	13.58	65	6.56	4.86	73.9	[9]
PANI/MoS_2_	Composite	~63	0.799	17.93	63	7.99	3.40	42.6	[12]
Pt	Photo-reduction	~95	0.810	13.53	66.6	7.29	5.85	80.3	[30]
Pt	Thermal decomposition	~80 ^a^	0.757	15.00	70.7	8.02	4.43	55.2	[30]
Pt	Polyol reduction ^b^	~80	0.702	13.77	67.6	6.55	5.01	76.6	-

^a^ Transmittance was calculated from absorbance. ^b^ Our work.
